# Oral Microbiome and SARS-CoV-2: Beware of Lung Co-infection

**DOI:** 10.3389/fmicb.2020.01840

**Published:** 2020-07-31

**Authors:** Lirong Bao, Cheng Zhang, Jiajia Dong, Lei Zhao, Yan Li, Jianxun Sun

**Affiliations:** ^1^State Key Laboratory of Oral Diseases, National Clinical Research Center for Oral Diseases, West China Hospital of Stomatology, Sichuan University, Chengdu, China; ^2^Department of Pulmonary and Critical Care Medicine, West China Hospital, Sichuan University, Chengdu, China; ^3^Department of Periodontics, West China Hospital of Stomatology, Sichuan University, Chengdu, China; ^4^Department of Cariology and Endodontics, West China Hospital of Stomatology, Sichuan University, Chengdu, China

**Keywords:** oral microbiome, SARS-CoV-2, influenza, microaspiration, co-infection, oral care

## Abstract

The new coronavirus SARS-CoV-2, the cause of COVID-19, has become a public health emergency of global concern. Like the SARS and influenza pandemics, there have been a large number of cases coinfected with other viruses, fungi, and bacteria, some of which originate from the oral cavity. *Capnocytophaga*, *Veillonella*, and other oral opportunistic pathogens were found in the BALF of the COVID-19 patients by mNGS. Risk factors such as poor oral hygiene, cough, increased inhalation under normal or abnormal conditions, and mechanical ventilation provide a pathway for oral microorganisms to enter the lower respiratory tract and thus cause respiratory disease. Lung hypoxia, typical symptoms of COVID-19, would favor the growth of anaerobes and facultative anaerobes originating from the oral microbiota. SARS-CoV-2 may aggravate lung disease by interacting with the lung or oral microbiota via mechanisms involving changes in cytokines, T cell responses, and the effects of host conditions such as aging and the oral microbiome changes due to systemic diseases. Because the oral microbiome is closely associated with SARS-CoV-2 co-infections in the lungs, effective oral health care measures are necessary to reduce these infections, especially in severe COVID-19 patients. We hope this review will draw attention from both the scientific and clinical communities on the role of the oral microbiome in the current global pandemic.

## Introduction

Corona Virus Disease 2019 (COVID-19) caused by a novel coronavirus, severe acute respiratory syndrome coronavirus 2 (SARS-CoV-2), have spread worldwide (Zhu N. et al., 2020). As of June 19, 2020, World Health Organization (WHO) reported over 8.3 million total confirmed cases and 450 thousand deaths of COVID-19 all over the world (World Health Organization, 2020), making it a severe threat to public health. However, the mechanism involving virus proliferation and how the virus interacts with other microorganisms in the lung is still unclear. The development of metagenomic next-generation sequencing (mNGS) technique allows for the investigation of novel or mixed pathogens (i.e., RNA viruses, DNA viruses, bacteria, and fungi) directly from original clinical samples (Shi et al., 2018), especially those from emergent patients. Metatranscriptome sequencing for the bronchoalveolar lavage fluid (BALF) of 8 COVID-19 patients revealed the presence of elevated levels of oral and upper respiratory commensal bacteria (Shen et al., 2020). It is well-known that an oral-lung aspiration axis is a key factor leading to many infectious diseases (Mammen et al., 2020).

The oral cavity houses the second largest microbiota in the human body and can include bacteria, fungi, viruses, and archaea (Dewhirst et al., 2010). As a large number of opportunistic pathogens and a variety of antibiotic resistance genes have been detected in the calculus of ancient human remains by analyzing the microbial DNA contained within them, it is supposed that the oral cavity, an ecological reservoir for potential pathogens, is involved in local and systemic diseases (Warinner et al., 2014). The main bacterial genera, by abundance, present within the normal oral cavity include *Neisseria*, *Corynebacterium*, *Leptotrichia*, *Streptococcus*, *Prevotella*, *Veillonella*, *Fusobacterium*, and *Capnocytophaga* (Li et al., 2014). The dominant genera of healthy lungs include *Streptococcus*, *Fusobacterium*, *Pseudomonas*, *Veillonella*, *Prevotella*, and *Capnocytophaga* (Beck et al., 2015; Dickson and Huffnagle, 2015; Wypych et al., 2019; Shen et al., 2020), colonizing the oral cavity as well. Microbiota that have migrated from the oral cavity, rather than those from the nasal flora, is likely to be an important source of microbiota in the lungs under normal conditions (Bassis et al., 2015). Multiple studies have shown that lung microbiota are more similar to those in the oropharynx, than those in the air, nasopharynx or lower digestive tract (Segal et al., 2013; Venkataraman et al., 2015). It was demonstrated that acellular bronchoalveolar lavage samples from half of healthy individuals are enriched with oral taxa (Segal et al., 2016), providing further evidence for the relationship between the microbial community in the lungs and the oral cavity. Additionally, many human bacterial pathogens can also colonize the respiratory tract of healthy individuals asymptomatically (Arora et al., 2015). The oral microbiome could be a driving force for bacteriome shifts by regulating mucosal immunity, which directly or indirectly affects pathogenicity (Li et al., 2019).

SARS-CoV-2 is a single-stranded RNA β-coronavirus and a member of the subgenus Sarbecovirus (beta-CoV lineage B). It is the seventh member of the *Coronaviridae* known to infect humans so far, two of which, SARS-CoV and MERS-CoV, caused global health threats previously (Guarner, 2020; Letko et al., 2020). Genome sequencing demonstrated that SARS-CoV-2 shares 79.5% sequence identity with SARS-CoV (Zhou et al., 2020), which is composed of single-stranded RNA with possible variations like those of the influenza virus. Other research has indicated a higher viral load in the nose than in the throat, viral nucleic acid change model in the patients infected with SARS-CoV-2 were similar to those in the patients with influenza viruses but different from that of SARS-CoV (Wang et al., 2020).

Following several influenza virus pandemics and the maturation of genome sequencing in recent years, studies on the co-infections caused by viruses, bacteria, or fungi have increased. Epidemiological data indicated that bacterial complications further increased morbidity and mortality of influenza infection. The “Spanish flu” of 1918 was the most severe influenza pandemic on record, 95% of the mortality due to bacterial infection (Morens et al., 2008). During the 2009 influenza pandemic, almost one in four patients presented bacterial complications (MacIntyre et al., 2018). The frequency of influenza and bacterial co-infection ranged from 2% to 65%. Among them, *Streptococcus pneumoniae* and *Staphylococcus aureus*, which accounted for 35% and 28% of infections (Klein et al., 2016). In addition, bacterial co-infection occurred in 7% of hospitalized COVID-19 patients. Compared with patients in mixed wards/intensive care unit (ICU) settings, ICU patients have a higher proportion of bacterial infections (Lansbury et al., 2020). These studies suggested that keen vigilance should be maintained against infections derived from the oral microbiome during infection by respiratory viruses and have uncovered risk factors such as increased inhalation, poor oral hygiene, and viral infection are related to the occurrence of respiratory infection (Pace and McCullough, 2010; Krone et al., 2014; Iki et al., 2016). The mechanisms by which the oral microbiome can influence respiratory disease is complicated and multifactorial, simultaneously affected by environmental, host, and microbial factors (Xu et al., 2018).

Considering the presented discussion, microbial co-infection increases the risk of disease severity in humans, while the mechanism is still unclear. Therefore, it is crucial to study the source and mechanism of co-infectious pathogens. In this review, we described the pathogens, i.e., viruses, bacteria and fungi, involved in co-infections in COVID-19 patients and summarized previous studies on the SARS-CoV and the influenza virus. We also discussed the possible disruptive factors contributing to co-infection and emphasized the potential role of the oral microbiome in the prognosis of COVID-19 patients. Understanding that the microbiome correlates with co-infections or affects the mucosal immune system helps to better predict the clinical outcomes and prevent complications.

## Co-Infection of Oral Microbiome and Sars-CoV-2 in the Lungs

It’s reported that 1450/2010 (72%) of COVID-19 patients received antimicrobial therapy (Rawson et al., 2020). The prevention and treatment of bacterial and fungal co-infections by antimicrobial prescribing in hospitalized patients with COVID-19 is necessary (Rawson et al., 2020; Verweij et al., 2020). There have been some reports on the microorganisms associated with infection, but the pathogenesis and effects are not fully understood. *Capnocytophaga*, *Veillonella*, and other oral opportunists have been found in the BALF of the COVID-19 patients by mNGS (Wu et al., 2020), indicating the oral cavity is likely to be a natural reservoir for pathogens inducing those found in COVID-19 patients with co-infections. Metagenomic sequencing has found that the nasopharyngeal bacterial population of COVID-19 patients varied with the length of viral infection. For example, *Fusobacterium periodonticum* significantly decreased 3 days after infection (Moore et al., 2020). As shown in the gut microbiota of patients with COVID-19 (Zuo et al., 2020), specific intestinal microbes that can downregulate the expression of ACE2 correlated inversely with SARS-CoV-2 load. Whether *Fusobacterium periodonticum* has the same function, further research should be done.

### The Mixed Infections in COVID-19

The majority of COVID-19 patients present a mild course, common symptoms including low fever, fatigue, and dry cough, while severe cases always present dyspnea within a week from the onset of symptoms, with rapid deterioration into serious complications, such as acute respiratory distress syndrome (ARDS), multiple organ failure, etc (Sharma et al., 2020). It is also notable that in some patients with no symptoms or negative RT-PCR results that chest computed tomographic (CT) scans revealed viral pneumonia-like lesions in the lungs, such as bilateral patchy shadows or ground glass opacity, and even lung consolidation, but the frequency is unknown (Wang et al., 2020; Yan et al., 2020).

During a multi-center study in Eastern China (Ai et al., 2020), nasopharyngeal swab samples were collected for total RNA extraction, followed by multiplex PCR and mNGS analysis. According to the results, 11 of 20 co-infections were identified in laboratory-confirmed COVID-19 cases and included regular respiratory viruses, fungi and bacteria. The most common pathogens detected were *Acinetobacter baumannii*, *Klebsiella pneumoniae*, *Aspergillus flavus*, *Candida glabrata*, and *Candida albicans*. In addition, 5 co-infection cases were found coinfected with a virus, including rhinovirus/enterovirus, H3N2, influenza B and respiratory syncytial virus, suggesting that virus co-infection were not uncommon in winter pneumonia patients. The sequencing revealed that *Leptotrichia buccalis*, *Veillonella parvula*, *Capnocytophaga gingivalis*, and *Prevotella melaninogenica* were overrepresented in the BALF of a COVID-19 patient (Wu et al., 2020). In another study (Ren et al., 2020), nucleic acid from the BALF of 5 hospitalized patients was extracted for deep sequencing. Those results showed that apart from SARS-CoVs, some bacterial pathogens could also be detected, including *Acinetobacter*, *Pseudomonas*, *Escherichia*, *Streptococcus*, and *Lactococcus*. Chen L. et al. (2020) reported that *Capnocytophaga* and *Veillonella* were found in the BALF of COVID-19 patients by mNGS. A recent study revealed coinfection with *Moraxella catarrhalis* in laboratory-proven cases of SARS-CoV-2, and quantified increased bacterial burden in some patients over others (Peddu et al., 2020). Most of above mentioned microbiota can be found in human oral cavity (Dewhirst et al., 2010), with the elevated level of oral commensal bacteria were found from the BALF of COVID-19 patients (Shen et al., 2020), it’s worth noting the co-infection occurs between oral microbiota and SARS-CoV-2 in the patients’ lungs.

SARS-CoV-2 mainly infect alveolar epithelial type II (AE2) cells (Zhao et al., 2020), the receptor for SARS-CoV-2, human angiotensin-converting enzyme 2 (ACE2), is also expressed in 0.52% of oral cells, of which 95.86% is expressed in tongue epithelial cells (Xu H. et al., 2020). This new evidence reveals that SARS-CoV-2 might interact with members of the oral microbiome in either the lungs or the oral cavity.

### The Bacterial Complications in Other Respiratory Viral Infections

In fact, bacteria are known to participate in interactions between host cells and viruses. Although bacteria are typically secondary invaders during influenza infections, they express virulence factors that promote viral pathogenesis. As a result, viral load increases and clearance rates decline (McCullers, 2014). Reviewing viral epidemics over the last two decades, such as the outbreaks of Severe Acute Respiratory Syndrome (SARS) in 2003 and H1N1 influenza in 2009, we found that the majority of deaths were caused by bacterial complications (Wang et al., 2003; Zheng et al., 2003; Li et al., 2005; MacIntyre et al., 2018). Long-term bed rest, mechanical ventilation, and glucocorticoid therapy are all risk factors for opportunistic infection. Long-term bed rest people cannot cough as easily or as well, which allows pooled mucus to stagnate and reduces the clearance of potentially pathogenic material and irritants (Knight et al., 2009).

Studies on confirmed SARS cases showed that co-infection was one of the major complications, especially in those who underwent extended hospitalization and long-term mechanical ventilation. Some conditional pathogens such as *Enterococcus faecalis*, *Klebsiella pneumonia*, *Acinetobacter baumannii*, *Stenotrophomonas maltophilia*, and *Candida albicans* could come from hospital environment, nosocomial infection, but the majority inhabit the oral cavity (Zheng et al., 2003; Li et al., 2005). Even though NGS technology was just coming into use during the SARS outbreak, it was difficult to identify the total microbial composition in clinical samples, therefore microorganisms from clinical specimens, such as blood, urine, sputum and BALF, of SARS patients were collected with conventional culture, and then underwent sensitivity testing and drug resistance testing (Zhang et al., 2004; Li et al., 2005). Some pathogens, such as anaerobes, cannot be easily cultured, making accurate diagnosis and treatment of infections challenging.

Influenza is the most common type of acute respiratory infection (Hanada et al., 2018) and bacterial complications were an important complication of the 2009 influenza pandemic (MacIntyre et al., 2018). Similar to SARS, conditional pathogens were the most common infectious microbes (Muscedere et al., 2013; MacIntyre et al., 2018). Conditional pathogenic bacteria, such as *Legionella pneumophila* (Rizzo et al., 2010), *Neisseria meningitidis, Moraxella catarrhalis* (Dela Cruz and Wunderink, 2017), from the upper respiratory tract are also associated with influenza co-infection. Furthermore, comprehensive detection of potential pathogens was performed using next-generation DNA sequencing of total RNAs extracted from an autopsied lung of a patient died of A/H1N1/2009. *Streptococcus pneumoniae* and *Porphyromonas gingivalis* were detected, though in small quantities. *P. gingivalis* is the most important periodontitis pathogen, while *Streptococcus* sp., in general, constitute the main component of oral flora (Kuroda et al., 2010). All of these indicate a myriad of relationships between viral infection and the oral microbiome.

### How the Oral Microbiome May Cause Lung Infection

Risk factors such as poor oral hygiene, cough, increased inhalation under normal or abnormal condition, and mechanical ventilation are the main routes by oral microbiota enter the airways. Additionally, lung hypoxia would stimulate the growth of anaerobes and facultative anaerobes derived from oral microbiota. Together, these factors result in respiratory dysbiosis, and thus cause respiratory disease.

#### Oral Hygiene

An increasing number of research projects aim to assess oral health and the prevalence of pre-existing oral colonization by respiratory pathogens in the elderly, and to verify whether these factors could influence pneumonia progression. Various types of oral microorganisms can be detected from the lungs due to different risk factors. Periodontal pathogens such as *Treponema denticola, P. gingivalis, Fusobacterium nucleatum, Actinobacillus actinomycetemcomitans* and *Veillonella parvula*, have been detected in the lungs of ICU patients (Kumar, 2017; de Carvalho Baptista et al., 2018). The tongue dorsum is one habitat niche of oral microbes, there the dominant microflora are *Prevotella* and *Veillonella*, which are associated with an increased risk of death due to pneumonia in older, frail patients (Asakawa et al., 2018; Kageyama et al., 2018). Dental plaques contain pathogens which could aggravate the state of pneumonia (Olsen, 2015), including *P. gingivalis*, which can synergistically enhance pathogenicity (Miller et al., 2018). Poor oral hygiene habits can lead to the accumulation of many periodontal microorganisms in the oral cavity, and oral dysbiosis can accelerate lung function decline (Takeuchi et al., 2018; Winning et al., 2019), which in turn can increase the incidence of pneumonia (Kumar, 2017). A one-year longitudinal study of 60 dependent elderly showed that the amount of respiratory pathogens colonizing the tongue and calculus, e.g., *P. aeruginosa* and *H. influenzae*, were high risk factors for pneumonia progression, while oral hygiene measures, such as removal of calculus and tongue biofilm, helped to reduce this (Hong et al., 2018). In conclusion, oral hygiene measures are necessary to protect people who are vulnerable to respiratory infections.

#### Cough and Inhalation

Because the dominant bacteria in various ecological niches of the body are different, microaspiration and transcolonization can result in systemic dysbiosis and disorders. Oral microorganisms reach the lower respiratory tract primarily through these mechanisms (Soussan et al., 2019) in severe patients located in hospitals and sanatoriums, or elderly people with dysphagia. A half-year prospective cohort study (Man et al., 2019) showed that nasopharyngeal microecological imbalance was caused by transcolonization of oral microbiota, leading to upper respiratory tract infections. It is speculated that aspiration pneumonia is also closely related to oral microorganisms due to the aspiration of oropharyngeal secretions (Hajishengallis, 2015). The frequency of microaspiration will rise in chronic inflammatory respiratory diseases, therefore it is necessary to pay attention to co-infections caused by oral microorganisms.

#### Mechanical Ventilation

Mechanical ventilation is generally used to assist or replace spontaneous breathing, involving two types, invasive ventilation and non-invasive ventilation. The former refers to mechanical ventilation that involves any instrument entering the trachea through the oral cavity, like trachea intubation and ECMO. Non-invasive ventilation, such as face or nasal masks, are generally appropriate for mild illnesses. Ventilator-associated pneumonia (VAP) frequently occurs in patients requiring mechanical ventilation, leading to high mortality, with an incidence from 5% to 67% depending on the population studied and the diagnostic criteria used (Barbier et al., 2013; Torres et al., 2017). In the process of orotracheal intubation, bacteria can rapidly migrate from the oral cavity and upper respiratory tract into the lungs (de Carvalho Baptista et al., 2018). Another risk factor is that ICU mechanical ventilation patients find it difficult to clear oral secretions via swallowing or coughing (Paju and Scannapieco, 2007). Many studies have reported that the main pathophysiologic mechanism for VAP development is oropharyngeal colonization (Doré et al., 1996; Bahrani-Mougeot et al., 2007). Several members of the oral microbiota (e.g., *Streptococcus oralis, Prevotella salivae*, and *Mycoplasma salivarium*) have previously been detected by 16S rRNA gene analysis in the BALF samples of VAP patients (Bahrani-Mougeot et al., 2007). Respiratory distress and hypoxemia are the main clinical symptoms in severe COVID-19 patients due to serous exudation and hyaline membrane formation of their alveolar spaces. Oxygen saturation, as measured in their fingers, of less than 93% or as low as 50% have been observed (National Health Commission of the People’s Republic of China, 2020). Therefore, they require high-flow nasal cannula or higher-level oxygen support measures to correct this hypoxemia. The lung hypoxia created in these patients is a suitable environment for the growth of anaerobic bacteria including those from the oral microbiota. Therefore, more attention should be paid to potential infections arising from oral microbiota before, and during, mechanical ventilation.

## Interaction Between Oral Microbiome and Virus Infection

SARS-CoV-2 can be detected from different body fluids and the rate of positive test results vary depending on the sample size, days after illness onset, and severity of illness. According to a study on 213 confirmed COVID-19 patients, viral RNA could be detected in all BALF samples of severe cases, but not from mild cases. Sputum appeared to be a good clinical sample with a high positive rate (74.4∼88.9%), followed by nasal swabs (53.6∼73.3%) and throat swabs (50∼ 61.3%). When the onset time was greater than 14 days, the positive rate of BALF, sputum, and nasal swabs showed similar results, and were still much higher than throat swabs (Yang et al., 2020). The viral load in throat swabs and sputum samples peaked at 5–6 days after onset and ranged from 10^4^ to 10^7^ copies/ml (Pan et al., 2020). What’s more, saliva appeared to be a good clinical specimen in the early stage of the disease, with SARS-CoV-2 detected in the initial saliva of 91.7% (11/12) of patients (To et al., 2020b), however the sample size was small in that study. Another study (To et al., 2020a) reported that the viral load of SARS-CoV-2 in posterior oropharyngeal saliva samples was highest during the first week of symptom onset. SARS-CoV-2 show high affinity with ACE2, which are higher in tongue, than in buccal or gingival, tissues (Xu H. et al., 2020).

At present, the impact of coronavirus on the oral, lung, and gut microbiomes has not been well-researched. However, we found a study on the changes in gut microbiota of swine infected by porcine epidemic virus (a member of the family *Coronaviridae*). Interestingly, *Fusobacteria*, a typically dominant taxa in the oral cavity, was found to predominate in the infected group (approximately 32%, 0.1% in the normal group) (Koh et al., 2015). Due to the limited amount of data available to date, we would like to focus on the influenza virus and discuss the relationship between influenza viruses and the oral microbiome. It was found that over-expressed *Prevotella* proteins can promote viral infection. As per the results, *Prevotella* proteins, rather than viral proteins, are involved in increasing clinical severity of COVID-2019, i.e., *Prevotella* plays role in COVID- 2019 outbreak and attention should be paid to understanding disease mechanisms and improving treatment outcomes (Khan and Khan, 2020).

### Oral Microbes Affect Influenza Viral Neuraminidase Activity

Influenza viruses rely on neuraminidase (NA) activity to release progeny viruses from infected cells and spread the infection. NA is, therefore, an important target of anti-influenza drugs. It was known that the culture supernatants of NA-producing oral bacteria, like *Streptococcus oralis* and *Streptococcus mitis*, can promote the release of influenza virus and cell-to-cell spread of the infection, and also help to increase viral M1 protein expression levels and activation of cellular ERK. These effects were not observed with culture supernatants of *Streptococcus sanguinis* which cannot produce NA (Kamio et al., 2015). These findings suggest that an increase in the number of NA-producing oral bacteria could elevate the risk of and exacerbate the influenza infection. Considering the tendency of the virus to accelerate the progression of respiratory disease, it is worth noticing the potential impact of oral microbiota on respiratory viral infections. However, the mechanism of interaction between oral bacteria and respiratory viruses to promote lung infection has not been studied in depth, and further attention is urgently needed in this field.

### Influenza Virus-Drive Oral Microecological Imbalance

Previous research has shown that viruses can drive changes in both local microbial composition and quantity in the lungs. Leung et al. (2013) conducted an unbiased high-throughput sequencing method to analyze the oropharyngeal microorganisms of pneumonia patients with or without influenza virus infection, and found that the number of *Pseudomonas* and *Bacillus* in the oropharynx increased significantly after influenza virus infection, whereas the number of *Prevotella, Veillonella*, and *Neisseria* decreased significantly. A clinical study (Lu et al., 2017) showed a large number of *Streptococcus, Actinomyces* and *Rothia* colonizing the oropharynx of flu patients suffering from bacterial complications. What’s more, the imbalance of microbiota was also found in patients infected by SARS-CoV-2, *Fusobacterium periodonticum* significantly decreased in the nasopharynx (Moore et al., 2020),while in the gut, the proportion of probiotics (like *Bifidobacterium*, *Lactobacillus*, and *Eubacterium*) was significantly reduced, and the proportion of conditioned pathogenic bacteria was significantly increased, such as *Corynebacterium* and *Ruthenibacterium* (Yu et al., 2020), which could lead to microbial translocation and a second infection.

### Co-infection With *P. gingivalis* and Influenza Virus Increased Significant Apoptosis and Necrosis

As an opportunistic pathogen, *P. gingivalis* cannot be completely eliminated by host cells. An *in vitro* study suggested that during co-infection of *P. gingivalis* and influenza virus, significant apoptosis and necrosis can be observed in lung epithelial cells within a short timeframe (Connolly et al., 2017) as the Bcl2/Bax/Caspase3 signaling pathway was activated (Chen et al., 2018). Cytotoxic products of *P. gingivalis* (e.g., lipopolysaccharide) can induce host cells release of inflammatory cytokines such as TNF-α, IL-1β, and IL-6 (Ke et al., 2016), which in turn increase NO production leading to lung epithelial cell damage (Alayan et al., 2006; Wu et al., 2016; Ahamed et al., 2017).

## Effects of Virus and Oral Microbiome on Host Immunity

### Change of Cytokines

Like SARS-CoV and MERS-CoV, SARS-CoV-2 can also induce excessive and aberrant non-effective host immune responses that are associated with severe lung pathology, and lead to death (Hui and Zumla, 2019; Huang et al., 2020). Most moribund COVID-19 patients are suffering from a cytokine storm (Zumla et al., 2020), which is manifested by the increased plasma concentrations of IL-2, IL-7, IL-10, G-CSF, CXCL10, CCL2, CCL3, and TNF-α (Hui and Zumla, 2019; Huang et al., 2020; Li G. et al., 2020). It has been confirmed that the cellular immunity and cytokines status are closely related to the state of illness, with high levels of IL-6 and IL-10 levels in severe patients (Wan et al., 2020). We speculate that changes in cytokines reflect disease status to some extent.

The presence of oral microbes such as *Streptococcus, Prevotella* and *Porphyromonas* can not only change the microbial composition of the respiratory system, but also promote a series of cytokine responses and affect the immune homeostasis of the lungs. The levels of serum IL-6 and IL-8 were significantly increased in patients with lung dysfunction, and local inflammatory factors spread into systemic circulation (Yang et al., 2018). Under certain conditions, *Streptococcus gordonii* can attack host fibronectin, and subsequent cytokine production can induce inflammatory responses (Erb-Downward et al., 2011; Liu et al., 2018). The cell-wall-anchored protein Staphylococcal surface protein A (SspA), a key factor in regulating the host innate immunity, is an immunostimulatory component of *S. gordonii* that promotes bacterial adhesion, and purified SspA can induce IL-6 and monocyte chemotatic protein-1 production from human lung epithelial cells (Andrian et al., 2012).

*Prevotella* from microaspiration, can also participate in the immune homeostasis of the respiratory tract (Huffnagle et al., 2017). Lung inflammation has been associated with enrichment of the lung microbiome with *Prevotella*, this organisms primarily activates toll-like receptor 2 and enhance the expression of inflammatory cytokines, including IL-23 and IL-1 (Segal et al., 2013, 2016). *In vitro* experiments have shown that *Prevotella* can stimulate the production of IL-8, IL-6, and CCL20 in lung epithelial cells, which promotes mucosal Th17 immune response and neutrophil recruitment (Larsen, 2017).

Additional research has shown that *Porphyromonas* is likely to affect the immune response as well. Animal experiments (Benedyk et al., 2016) suggested that *P. gingivalis* can produce Gingipain, which then affect the innate immune response and promote chronic inflammation. Pathological manifestations such as intrapulmonary hemorrhage, necrosis, and neutrophil infiltration occur after infection with *P. gingivalis*. Lung tissue damage is associated with systemic inflammatory response, manifested by elevated levels of TNF, IL-6, IL-17, and C-reactive protein, and these pathological lesions are significantly dependent on the activity of *P. gingivalis*. Besides colonizing the lungs themselves, DNA from oral microbes can be transmited from the gingival tissue to the pulmonary vasculature (Chukkapalli et al., 2018), this might be a form of distant dissemination causing lung diseases.

### T Cell Responses to Infections

T cells are a type of lymphocyte which reside in the lungs and play a key role in protecting against chronic inflammation. Both the regulatory T cells (T-regs) and T helper cells (Th cells) are important defenders in the immune system. Previous research has shown that a large number of anaerobic bacteria originating from the oral cavity are present in the lungs of pulmonary tuberculosis patients, and bacterial metabolites (short-chain fatty acids) were associated with both the oral anaerobic bacterial load (e.g., *Prevotella*) and T-regs response (Segal et al., 2017).

A study including 1,099 patients with laboratory-confirmed COVID-19 found that the peripheral blood lymphocyte count in severe patients was 95.5% (147/154), which was significantly higher than that of mild cases (Guan et al., 2020b). It confirmed that the cellular immunity is closely related to the state of illness. In severe patients, CD4^+^ T and CD8^+^ T levels presented as low, indicating that T cell subsets can be used as one of the bases to predict the transition from mild to severe (Wan et al., 2020). Based on anatomic reports (Xu Z. et al., 2020), found that both the peripheral CD4^+^ T and CD8^+^ T counts of the COVID-19 patients were significantly reduced, but hyperactivated. Additionally, there was an increase in highly pro-inflammatory CCR6^+^ Th17 in CD4^+^ T cells and a high concentration of cytotoxic granules in CD8^+^ T cells. These results imply that hyperactivation of T cells, manifesting as the increase of Th17 and high cytotoxicity of CD8^+^ T cells, led to the severe immune injury of the patient to some extent.

### The Host Factors

SARS-CoV-2 is more likely to affect middle-aged and elderly people, especially those with comorbidities, as a result of the weaker immune functions of these patients (Chen N. et al., 2020). About 1/4∼1/2 patients infected by SARS-CoV-2 had chronic comorbidities, and the prognosis was strongly associated with the presence and number of comorbidities (Chen N. et al., 2020; Guan et al., 2020a). Malignancy and chronic obstructive pulmonary disease (COPD) appeared to be the main risk factors leading to poorer clinical outcomes, followed by diabetes and hypertension, according to a retrospective study of 1099 COVID-19 cases (Guan et al., 2020a). Therefore, immune disorders and long-term chronic inflammatory stimulation are likely to be key drivers to poor prognosis, however more mechanism studies are needed to confirm this.

#### Aging

Just like other chronic inflammatory conditions, it is crucial to bear in mind that the elderly, though perhaps seemingly healthy, might respond differently to infectious and inflammatory factors. Apart from the increased risk of severe disease, recovery could also be slower and poor, thus increasing the possibility of recrudescence or aggravation as time passes (Boe et al., 2017). As human beings age, the niche that *Streptococcus pneumonia (S. pneumoniae)* inhabit might change, resulting in a weakening of microbiome resiliency. This is related to the result of the degeneration of the immune system or a low-grade chronic inflammation (Krone et al., 2014). The phenomenon of decreased microbial diversity, in conjunction with the presence in the oral cavity of various groups of anaerobic commensals negatively related to the overgrowth of potential lung infection pathogens, was also observed among adults (de Steenhuijsen Piters et al., 2016). This not only suggest that these anaerobic commensals play an important role in the level of resilience against pathogen overgrowth and disease, but also emphasizes that the chronic inflammatory process contributes to the changes in microbiota composition, potentially making it harder to intervene.

#### Systemic Diseases Alter Composition and Diversity of the Oral Microbiome

Compared with healthy individuals, those with chronic diseases have a certain degree of microecological disorder. Here, we will review several comorbidities closely related to COVID-19 as examples to illustrate that changes in composition, quantity, and colonization sites may have potential effects on prognosis. Mounting evidence suggests that oral streptococci can be detected from the lungs of COPD patients (Pragman et al., 2018) with increased levels of *P. gingivalis* detected in the subgingival plaque (Tan et al., 2019). The oral microbiota of cancer patients has also been found to be significantly altered. *Veillonella*, *Streptococcus*, *Rothia* and *Aggregatibacter* were dramatically increased in non-small cell lung cancer patients (Zhang W. et al., 2019) and, except for *Prevotella*, several common oral genera (i.e., *Haemophilus, Neisseria* and *Streptococcus*) decreased in individuals with colitis-associated cancer (Flemer et al., 2018). Moreover, it has been reported that the diversity of oral microbiota in patients with diabetes decreases significantly, and is associated with an increase in the pathogenic content of the hyperglycemic microbiota (Saeb et al., 2019), such as *Capnocytophaga, Porphyromonas*, and *Pseudomonas* (Graves et al., 2019).

## Oral Care Interventions for Patients

Once infected by a virus such as SARS-CoV or SARS-CoV-2, patients are vulnerable to dyspnea, hypoxemia, and even acute respiratory distress syndrome (ARDS), and thus mechanical ventilation becomes a common life-saving intervention. Given the fact that oral microbiota is prone to entering the lower respiratory tract due to increased aspiration and the presence of mechanical ventilation, the prevalence of fatal VAP may increase. However, current guidelines for the management of COVID-19 scarcely emphasize oral care. As discussed above, poor oral hygiene can lead to a large accumulation of pathogens in the oral cavity and immune disorders caused by viral infection can promote additional bacterial/fungal infections, hence increase the risk of secondary pneumonia. Therefore, it is necessary for the clinician to strengthen oral hygiene care for COVID-19 patients.

Povidone iodine (PV-I, 0.23∼1%), cetylpyridinium chloride (CPC, 0.05∼0.10%), and hydrogen peroxide (0.5∼1.5%) all effectively reduce the number of oral microbiota (Ardizzoni et al., 2018; Eggers et al., 2018; Li and Meng, 2020). Studies have also shown that SARS-CoV, MERS-CoV, and influenza virus A (H1N1) can be efficiently inactivated by these compounds within 1 min (Kariwa et al., 2006; Eggers et al., 2015, 2018; Kampf et al., 2020; Li and Meng, 2020). Mouthwash containing chlorhexidine (CHX, 0.02%) or CPC have the ability to impair both the biofilm formation of *viridans streptococci* and the adhesion, proinflammatory effects, and immune escape abilities of *Candida albicans* (Ardizzoni et al., 2018). In addition, 0.05∼0.1% CPC showed potent, rapid activity against MERS-CoV (Shen et al., 2019) and influenza viruses (Popkin et al., 2017), however it’s activity against SARS-CoV-2 is currently unknown. Since SARS-CoV-2 is vulnerable to oxidation, antiseptic mouthwash or gel containing broad spectrum oxidative agents, such as hydrogen peroxide or PV-I, is recommended to reduce the oral microbiota and potentially SARS-CoV-2 as well. 0.02% of CHX is ineffective against coronaviruses as a surface disinfectant, it works as good as 7.5% povidone-iodine or 70% ethyl alcohol when the concentration was increased to 0.05% (Chin et al., 2020) (Table 1).

**TABLE 1:** Characteristics of four common components of mouth rinses.

Mouth rinses	Characteristics	Coronaviruses sensitive	Sources
Povidone iodine (PV-I, 0.23∼1%)	Reduce the number of oral microbiota. Inhibit the biofilm formation of *viridans streptococci* and the adhesion, proinflammatory effects, and immune escape abilities of *Candida albicans*	SARS-CoV, MERS-CoV and potentially SARS-CoV-2 sensitive	Kariwa et al., 2006; Eggers et al., 2015, 2018; Ardizzoni et al., 2018; Kampf et al., 2020; Li and Meng, 2020
Cetylpyridinium chloride (CPC, 0.05∼0.10%)	Reduce the number of oral microbiota. Inhibit the biofilm formation of *viridans streptococci* and the adhesion, proinflammatory effects, and immune escape abilities of *Candida albicans*	SARS-CoV, MERS-CoV sensitive. SARS-CoV-2 sensitivity is currently unknown	Kariwa et al., 2006; Eggers et al., 2015, 2018; Ardizzoni et al., 2018; Shen et al., 2019; Kampf et al., 2020; Li and Meng, 2020
Hydrogen peroxide (H_2_O_2_, 0.5∼1.5%)	Reduce the number of oral microbiota	SARS-CoV, MERS-CoV and potentially SARS-CoV-2 sensitive	Kariwa et al., 2006; Eggers et al., 2015, 2018; Ardizzoni et al., 2018; Kampf et al., 2020; Li and Meng, 2020
Chlorhexidine (CHX, 0.02∼0.05%)	Reduce the number of oral microbiota. Inhibit the biofilm formation of *viridans streptococci* and the adhesion, proinflammatory effects, and immune escape abilities of *Candida albicans*	0.02% CHX is ineffective against coronaviruses while 0.05% is SARS-CoV-2 sensitive	Ardizzoni et al., 2018; Chin et al., 2020

Several studies have reported that improving oral hygiene care by either mechanical or chemical control of dental plaque biofilm formation can reduce the number of potential respiratory pathogens and consequently the risk and mortality risk of aspiration pneumonia by as much as 60% (Dennesen et al., 2003; Tuon et al., 2017). Oral hygiene care, in these cases, include the use of mouthwash or gels, toothbrush, alone or in combination, together with sputum aspiration and saliva maintenance (Hua et al., 2016). Specifically, the following practices should be taken: brushing of the teeth, gums, and tongue at least twice a day, moistening the oral mucosa and lips every 2–4 h, rinsing the mouth with mouthwash containing 0.5∼1.5% hydrogen peroxide, 0.05∼0.10% CPC or 0.23∼1% PV-I twice a day and wiping with 1.5% hydrogen peroxide.

## Research Approaches for Co-Infections

There are many key unsolved questions in the field of co-infections. A better understanding of the complex relationship between viruses, hosts, and bacteria will help us cope with common manifestations, such as community-acquired pneumonia, and help us prepare for the next severe pandemic. The study of lung organoidsand animal models of co-infection can help us to better study co-infection and push forward the field.

### Better Diagnostics of Co-infections

A variety of detection methods are designed to analyze the co-infection of new coronavirus and other microorganisms (Zhang et al., 2020). Fluorescence quantitative PCR (RT-PCR) detection of viral nucleic acid is the main method of identification (Hashemzadeh et al., 2016; Kim et al., 2016; Noh et al., 2017; Zhang H. et al., 2019). Of course, specific primers based on known bacterial/fungal sequences can be designed to detect co-infected species and multiplex RT-PCR was applied to seven family members for differential diagnosis of 18 types of viruses and 4 types of bacteria (Chan et al., 2020). Digital loop-mediated DNA amplification (LAMP) based on micro-nanofluidic chip (Gansen et al., 2012; Rems et al., 2016) can simultaneously detect several common viruses (Shirato et al., 2018). By collecting deep expectoration or BALF, mNGS can identify the main pathogen SARS-CoV-2 (Chen L. et al., 2020; Ren et al., 2020). It has also a high clinical value for the identification of the combined or secondary bacteria, especially for Critical Care patients with sepsis, immunosuppressive host complicate with severe infection, severe pulmonary infection, etc (McArdle et al., 2018). Multiple studies sequenced samples of COVID-19 suspected or confirmed patients found the presence of co-infection, such as oral bacteria, fungi, viruses, etc (Chan et al., 2020; Cox et al., 2020; Lansbury et al., 2020; Tadolini et al., 2020), which may come from the environment or colonize the mouth. As a result, once the doctors quickly and accurately find the main and sub-pathogen. They can develop drug treatment protocols targeting the pathogens and evaluate treatment effectiveness, and adjust treatment means for severe patients as early as possible.

### Lung Organoids

“Lung organoids” refers to self-assembled structures produced by three-dimensional cultured lung epithelial progenitor cells, with or without mesenchymal support cells (Barkauskas et al., 2017). Different epithelial cell populations of the adult lung, including basal cells, secretory Club cells and AEC2 cells, as well as human pluripotent stem cells (hPSCs), are the most attractive sources for lung organoids (Barkauskas et al., 2017). Over the past decade, they have become indispensable tools for basic research and translational research (Paolicelli et al., 2019). Organoid is a new “secret weapon” that enables precision medicine. Lung organoids could be developed to study the bacterial co-infection in patients with COVID-19.

Over the past decade, stem cell-derived, three-dimensional, self-organizing organoids have emerged as a new way to simulate respiratory diseases *in vitro* (Li Y. et al., 2020). With the help of cutting-edge technologies, like the high-throughtput organoid microinjector system (Williamson et al., 2018), microinjection of microorganisms into the organoid lumen can be realized to facilitate the study of host-microbial interaction in 3D structures (Bertaux-Skeirik et al., 2015). Nowadays, lung organoids have been used in microbial infection studies to understand the molecular mechanism of epithelial renewal after viral infection (Quantius et al., 2016), and to study the cytokine profile release when stimulated by *Pseudomonas aeruginosa* (Shen et al., 2017). The most difficult obstacle to overcome at present is the complete differentiation of hPSCs cultures into specialized lung cell types, especially AEC1 cells and alveolar-like cells. Efforts are needed to explore new classes of stem cells and support cells, quantify using organoid culture, and compare them with known populations to find better seed cells.

Due to the short-term organoids culture time (several weeks), lung organoids can be applied to studying the pathogenesis and mechanism of viral-bacterial co-infection or making medication guidelines. And a precise diagnostic platform can be developed based on organoids (Lamers et al., 2020). Furthermore, patient-derived organoids (PDO) has high predictive ability for co-infection drug candidates, and is suitable for high-throughput cultivation, and thus can be used as an effective drug screening platform (Vlachogiannis et al., 2018). Take full advantage of the powerful genomic editing technology of CRISPR/Cas9, especially to study the role of specific genes in the self-renewal and differentiation of human lung stem cells and the generation of human respiratory disease models. Organoids which can be modified by various genome-editing techniques and thus different genes can be constructed to express human ACE2 protein to study the pathogenesis of co-infection in COVID-19.

### Animal Models of Co-infection

According to the mouse construction strategies and methods, the current SARS-CoV-2 infected mouse model is divided into three types: (1) Using mouse hepatitis virus (MHV), a murine coronavirus, as model virus to study its genetic and biological mechanisms in mouse (Weiss, 2020); (2) Employing gene editing technology to knock out mouse genes such as *ace2* and *Tmprss2*, genes related to viral binding and entry, to explore the pathogenicity-related host factors (Iwata-Yoshikawa et al., 2019; Hoffmann et al., 2020), or transfer human protein expressing genes (i.e., human *ace2*) into mice to allow a direct infection of SARS-CoV-2 (Jiang et al., 2020); (3) Infecting mice with wild-type SARS-CoV-2 virus repeatedly to cultivate an adaptive version of the virus (Dinnon et al., 2020). So as to establish a mouse model of virus infection that can cause a significant clinical phenotype. Based on the establishment of SARS-CoV-2 single infected mouse model, a co-infection model could be explored by following inoculation of other pathogens, given the fact of viral-bacterial situation during COVID-19 pandemic (Zhu X. et al., 2020).

## Conclusion

**FIGURE 1:**
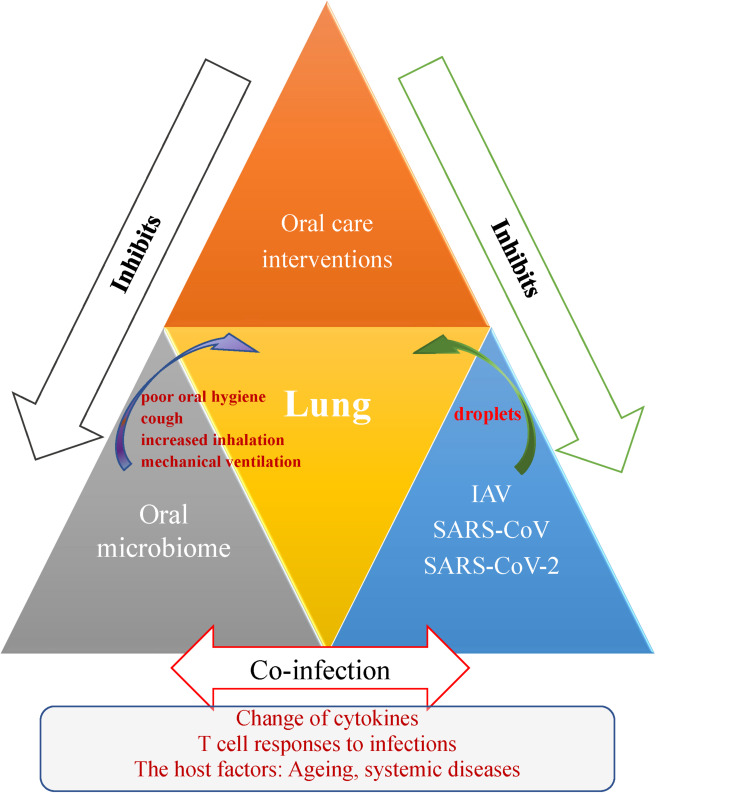
Schematic representation outlining the oral-lung-axis mechanism of viral-bacterial lung co-infection during respiratory viral infections and effect of the oral care interventions for patients. Risk factors such as poor oral hygiene, cough, increased inhalation and mechanical ventilation provide a pathway for oral microorganisms to enter the lower respiratory tract and thus cause co-infection to aggravate respiratory disease via mechanisms involving changes in cytokines, T cell responses, and the effects of host conditions such as aging and the oral microbiome changes due to systemic diseases. Improving oral hygiene care by either mechanical or chemical control of dental plaque biofilm formation would reduce the number of potential respiratory pathogens and inhibit droplet-borne virus route through oral cavity, and hence consequently reduce the risk and mortality risk of aspiration pneumonia.

In this review, we described the co-infections in viral disease. The mechanism of viral-bacterial lung co-infections during respiratory viral infections and effect of the oral care interventions for patients are shown in Figure 1. During the SARS-CoV-2 global outbreak, numerous cases are coinfected with other pathogens, some of which originate from the oral cavity. Until now, little work had been done on the coronaviruses and oral microbiomes, and much remains for future study. While previous research has linked lung regional immunity with stable state maintenance or imbalance, current research has only preliminarily explored the influence of oral bacteria in the respiratory system, or the influence of oral bacteria-virus interactions on respiratory diseases. The scientific community must help the world to prepare for the next pandemic and thus to avoid massive loss of life. Drawing on the experiences of SARS in 2003 and influenza outbreaks, we recommend that oral hygiene in COVID 19 patients be a concern as co-infection is critical to prognosis.

## Author Contributions

LB and CZ contributed to conception, design, and drafting the manuscript. JD and LZ contributed to conception. YL and JS contributed to conception, design, and critically revise the manuscript. All authors gave final approval and agree to be accountable for all aspects of the work.

## Conflict of Interest

The authors declare that the research was conducted in the absence of any commercial or financial relationships that could be construed as a potential conflict of interest.
